# Diazoxide‐Responsive Congenital Hyperinsulinism in a Preterm Infant With Homozygous *ABCC8* Mutation: A Case Report

**DOI:** 10.1155/crie/7968566

**Published:** 2026-09-23

**Authors:** Prerana Kansakar, Grisha Gurung, Sadikshya Bhandari, Kriti Shrestha, Bhumika G. C., Shreya Thapa, Rasik Acharya

**Affiliations:** ^1^ Department of Pediatrics, Patan Academy of Health Sciences, Lalitpur, Nepal, pahs.edu.np

**Keywords:** *ABCC8* gene, case report, diazoxide, DOPA PET CT scan, hyperinsulinism

## Abstract

**Introduction:**

Congenital hyperinsulinism (CHI) is characterized by inappropriate insulin secretion resulting in persistent neonatal hypoglycemia. CHI is often linked to mutations in the *ABCC8* and *KCNJ11* genes and can lead to persistent hypoglycemia, seizures, and neurological injury, making early diagnosis and management essential.

**Case Report:**

We describe a late‐preterm Nepalese infant born at 34 weeks and 5 days of gestation, weighing 5.26 kg, to a 32‐year‐old gravida four nondiabetic mother. The infant developed significant hypoglycemia just 2 h after birth. Despite a glucose infusion rate (GIR) of 12 mg/kg/min, the infant had persistent hypoglycemia. A critical sample confirmed hyperinsulinism, prompting treatment with octreotide, intravenous (IV) GIR of 17 mg/kg/min and later diazoxide. Due to limited IV access, a continuous enteral GIR of 9 mg/kg/min was administered through an orogastric (OG) tube. Genetic testing revealed a homozygous pathogenic p.Arg1214Gln mutation in the *ABCC8* gene, and ^18^F‐DOPA PET‐CT (2‐deoxy‐2‐[fluorine‐18]fluoro‐D‐glucose positron emission computed tomography) showed diffuse pancreatic uptake. At 5 months, interruption of diazoxide therapy led to a hypoglycemic seizure (blood glucose level: 29 mg/dL), which resolved after the treatment resumed. Despite the typical association of homozygous *ABCC8* mutations with diazoxide nonresponsiveness, this infant achieved sustained glycemic control on diazoxide. On subsequent follow‐ups, the child remains stable and continues to achieve normal developmental milestones.

**Conclusion:**

Early recognition and timely management are crucial in effective management of CHI. This case shows rare diazoxide responsiveness in homozygous *ABCC8* mutation. This case suggests that a carefully monitored therapeutic trial of diazoxide may still be reasonable in selected patients with homozygous *ABCC8* mutations before concluding complete medical unresponsiveness.

**Highlights**

• The neonate required prompt and aggressive treatment for CHI, including high glucose infusion rates, diazoxide, and octreotide.
• The baby showed a response to diazoxide and was managed with oral glucose infusion, effectively stabilizing blood glucose levels.
• Genetic testing identified a homozygous *ABCC8* mutation, and an ^18^F‐DOPA PET scan revealed a uniformly enlarged pancreas.
• By 15 months, the infant had normal developmental milestones and no further hypoglycemic episodes.

## 1. Introduction

Congenital hyperinsulinism (CHI) is characterized by excessive insulin secretion in the presence of low plasma glucose levels and is the most common cause of persistent and refractory hypoglycemia. Pathologically, it manifests as a diffuse or focal disease. In 30% to 66% of cases, mutations in particular genes (*ABCC8* or *KCNJ11*) encoding two subunits of the pancreatic beta‐cell ATP‐sensitive potassium (KATP) channel result in uncontrolled insulin release [1, 2]. The incidence ranges from 1/27000 to 1/50000 in newborns [3]. Prompt diagnosis and management of the underlying hypoglycemia disorder are critical for preventing brain damage and improving outcomes [4].

The mainstay of treatment with diazoxide and somatostatin analog (octreotide) is necessary in metabolic stabilization with increased glucose supply and decreased insulin production. If medical therapy fails to control the glycemic level, surgery is considered. Before determining the nature of the lesion, an ^18^F‐DOPA PET scan is done. Depending on whether it is focal or diffuse disease, the procedure is either excision of the lesion or subtotal pancreatectomy [3].

However, diazoxide responsiveness in homozygous *ABCC8* mutations remains exceptionally rare, with limited reported cases. This case presents the unique clinical course of a preterm neonate with a homozygous *ABCC8* mutation, demonstrating a positive response to diazoxide. Additionally, the use of an oral glucose infusion to stabilize blood glucose levels highlights an innovative approach to vascular access limitation in a resource‐limited setting.

## 2. Case Report

### 2.1. Patient Information

We report the case of a Nepalese male infant born at 34 weeks and 5 days of gestation via emergency cesarean section due to being large for gestational age (LGA). We evaluated this case at a tertiary care center in Lalitpur, Nepal, in 2024. The infant was born to nonconsanguineous parents. The mother is a 32‐year‐old gravida four with a normal prepregnancy body mass index (BMI) and no other medical conditions during her regular antenatal checkups with a normal oral glucose tolerance test. She had a prior undocumented induced abortion 5 years earlier due to fetal abnormalities, but her other children were born healthy at term. She had no pregestational or gestational risk factors for diabetes. Antenatal ultrasonography performed 12 days prior to delivery at 33 weeks showed a markedly increased estimated fetal weight of 3.787 ± 0.300 kg, which is well above the 99th percentile for gestational age (*z*‐score = + 4.14) and showed no anomalies.

### 2.2. Clinical Findings

The actual birth weight was 5.26 kg, which is >99th percentile. The infant cried immediately after birth, with APGAR scores of 8/10 at 1 min and 9/10 at 5 min. Physical examination revealed no gross dysmorphic features or signs of overgrowth syndromes like macroglossia, hemihyperplasia, or abdominal wall defects. The systemic examination was unremarkable.

### 2.3. Diagnostic Assessment

The infant was admitted to the nursery postdelivery for observation, with feeding initiated within the first hour of life. Glucose random blood sugar (GRBS) was monitored using a portable glucometer as per the standard LGA protocol. Readings were 50 mg/dL at 1 h and 55 mg/dL at 2 h of life (threshold for asymptomatic late‐preterm neonate < 4 HOL: <25 mg/dL) [5]. However, at 4 h of life, the GRBS dropped to 16 mg/dL, accompanied by lethargy and jitteriness, meeting criteria for symptomatic hypoglycemia (threshold: <50 mg/dL) [5].

In accordance with the management protocol for symptomatic hypoglycemia, an immediate dextrose bolus was administered, and hourly glucose monitoring was done. Despite systematic escalation of the glucose infusion rate (GIR), blood sugar levels remained consistently below 50 mg/dL with a laboratory‐confirmed serum glucose of 40 mg/dL on intravenous (IV) GIR of 10 mg/kg/min. The infant was therefore transferred to the neonatal intensive care unit (NICU), where an umbilical catheter was placed, and the GIR was gradually increased.

At 52 h of life, beyond the physiological transitional period, hypoglycemia persisted despite a GIR of 12 mg/kg/min. A critical sample was collected to investigate persistent hyperinsulinism and rule out other metabolic or endocrine causes (Table 1). Laboratory analysis confirmed hyperinsulinemic hypoglycemia (Table 2), and the lactate level was 1.1 mmol/L (normal: 0.8–1.4 mmol/L). These findings effectively ruled out adrenal insufficiency and lactic acidosis, establishing the diagnosis of hyperinsulinism.

**Table 1:** Critical sample of the patient.

Tests	1 DOL (done during hypoglycemic episode)	5 DOL (done during hypoglycemic episode)	During follow‐up on resolution of hypoglycemia	Biochemical method of analysis	Reference range
Cortisol	23.38	—	—	Electrochemiluminescence immunoassay (ECLIA)	6.2–19.43 mcg/dL
Insulin	33.18	78.75	5.63	ECLIA	2.6–37 mIU/mL (during normal glucose level) <2 mIU/mL (during hypoglycemia)
17‐Hydroxy progesterone	15.64	—	—	Chemiluminescence immunoassay (CLIA)	Up to 17.3 ng/mL
C‐peptide	—	7.26	—	ECLIA	0.010–40 ng/mL

**Table 2:** Laboratory monitoring during diazoxide therapy.

Parameters	Baseline lab value	After diazoxide therapy	Reference range
Serum albumin	3.1 g/dL	3.2 g/dL	2.8–4 g/dL
Urea	24 mg/dL	31 mg/dL	19–43 mg/dL
Creatinine	0.3 mg/dL	0.3 mg/dL	0.8–1.5 mg/dL
Sodium	134 mEq/L	138 mEq/L	135–145 mEq/L
Potassium	4.7 mEq/L	4.5 mEq/L	4.6–6.7 mEq/L
SGPT	41 U/L	57 U/L	10–40 U/L

### 2.4. Intervention

The GIR was increased to 17 mg/kg/min to meet the metabolic demand, and simultaneously, octreotide was started subcutaneously at 5 mcg/kg/day as diazoxide was not immediately available in Nepal. Octreotide was titrated up to a maximum dose of 25 mcg/kg/day in divided doses. A repeat evaluation revealed a high level of insulin (Table 1). After the diazoxide capsules were brought from India, they were taken to the hospital pharmacy and professionally compounded into a syrup for neonatal administration. Oral diazoxide was initiated at 5 mg/kg/day in three divided doses and was gradually titrated to 15 mg/kg/day.

An echocardiogram was performed, which identified a moderate‐size patent ductus arteriosus (PDA) measuring 3.2 mm with mildly dilated right atrium and right ventricle. There were no signs of hypertrophic cardiomyopathy. To manage the potential for fluid retention due to diazoxide use, hydrochlorothiazide was added to the treatment regimen at a dose of 5 mg/kg/day.

Due to limited IV access, a continuous oral GIR of 9 mg/kg/min was introduced via an orogastric (OG) tube using a syringe pump. Following the methodology of Vajravelu et al. [6], we prepared a 15% dextrose solution by mixing 25% and 10% dextrose in a 1:2 ratio (e.g., 50 mL of 25% + 100 mL of 10%), resulting in an approximate osmolarity of 750 mOsm/L, which remained within safe limits for continuous administration. We strictly monitored gastric residuals, abdominal distension, stool consistency, and blood glucose levels.

Once blood glucose stabilized, breast milk was administered via an OG tube every 2 h while the oral GIR was tapered. The infant successfully transitioned to direct spoon and bottle feeding. After stabilizing on oral feeds, octreotide was tapered and discontinued. Despite controlled GRBS and a normal metabolic panel, the neonate developed seizures, prompting the initiation of levetiracetam. A brain MRI was planned, but the parents declined due to financial issues.

USG cranium was performed at 7 days of life, at 28 days of life, and following the later episode of seizure; all three scans were normal. Abdominal and pelvic ultrasound (USG) revealed no abnormalities, but further imaging, including multidetector computed tomography (MDCT), showed mild hepatomegaly.

After 2 months of hospital stay, the infant’s blood glucose levels were stabilized on a combination of oral diazoxide and hydrochlorothiazide during this period, intensive training was given to the parents on home glucose monitoring using a portable glucometer and the correct administration of the compounded diazoxide syrup. About 6 h of safety fast was performed prior to discharge. The neonate was discharged on a maintenance regimen of diazoxide (15 mg/kg/day), hydrochlorothiazide (5 mg/kg/day), and levetiracetam for seizure prophylaxis.

At 5 months of age, the infant experienced hypoglycemic seizures. This was directly caused by the unavailability of diazoxide, leading to an interruption in treatment. At the time of the presentation, blood glucose was 29 mg/dL.

Neurological examination: Postictal, the infant was lethargic but showed no focal neurological deficits. Cranial nerve examination, muscle tone, and deep tendon reflexes were all within normal limits for the age. Although brain MRI could not be performed due to financial constraints, serial neurodevelopmental assessments remained normal during follow‐up.

**Figure 1:**
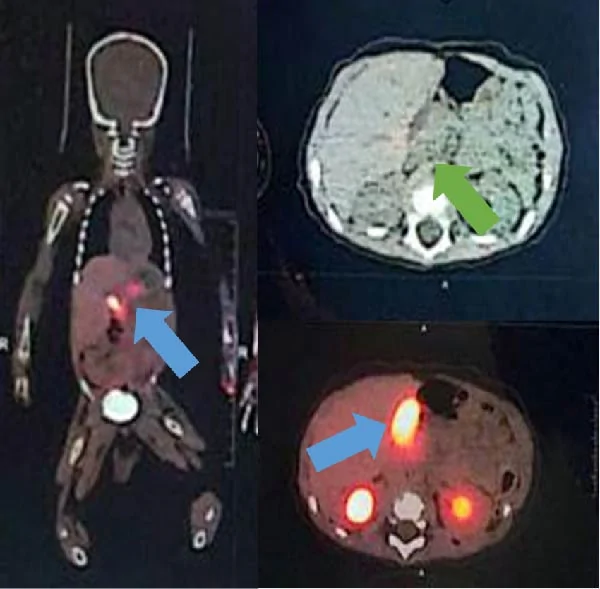
^18^F‐DOPA PET/CT demonstrating diffuse pancreatic uptake consistent with diffuse congenital hyperinsulinism.

**Figure 2:**
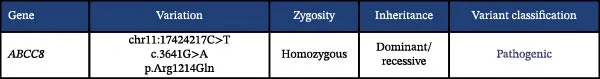
Genetic testing was positive for a homozygous “pathogenic” variant in exon 29 of *ABCC8* gene with variation chr11:17424217C>T c.3641G>A p.Arg1214Gln.

The infant was readmitted to the hospital and required an IV GIR for immediate stabilization. Once diazoxide was obtained, it was restarted at the initial dose of 15 mg/kg/day. IV GIR was slowly tapered and then discontinued as oral feeding was maintained. Throughout this admission, the infant remained playful, and the neurological examination was normal. A repeat echocardiography was performed, which was normal. The infant was discharged after 7 days. For further evaluation, the infant was referred to India at 8 months of life. An ^18^F‐DOPA PET scan was performed, which revealed a bulky pancreas with diffuse FDOPA uptake (Figure 1). Genetic testing confirmed a homozygous “pathogenic” variant in exon 29 of the *ABCC8* gene (chr11:17424217C >T; c.3641G >A; p.Arg1214Gln) using transcript NM_000352.3 (Figure 2).

The pathogenicity of this variant is supported by five out of five in silico prediction tools, including FATHMM, LRT, mutation assessor, mutation taster, and SIFT. Additional evidence includes a rare allele frequency of 0.0023% in the gnomAD database; however, no homozygosity for this variant has been identified. This classification fulfills the American College of Medical Genetics (ACMG) criteria (Table 3). Parents were advised to undergo genetic testing however, they declined due to financial issues.

**Table 3:** ACMG criteria.

ACMG criterion	Strength	Evidence
PM2	Supporting	Rare in gnomAD (0.005%, 1/18,394 East Asian alleles; dbSNP rs367850779) [7]
PM3	Strong	Out of five reported affected individuals (PMID: 9618169, 15562009, 20685672, 14692646, 9648840) two were compound heterozygous with another pathogenic *ABCC8* variant in trans (ClinVar Variation ID: 632619; trans allele Variation ID: 9088, 371380; PMID: 9648840, 14692646) [8–12]
PS3	Supporting	Shyng et al. [10] (1998, PMID: 9648840): reduced MgADP stimulation and diazoxide responsiveness
PM5	Supporting	Other pathogenic variant(s) affecting the p.Arg1214 residue have been reported (PMID: 17575084, 23275527, 24401662, 24937539, 26180531)—citation‐level confirmation from ClinVar; recommend independently verifying at least one source describes a distinct amino acid substitution at this residue before finalizing [13–17]
PP2	Supporting	*ABCC8* has a low rate of benign missense variation, and missense variants are a well‐established disease mechanism for *ABCC8*‐related congenital hyperinsulinism
PP3	Supporting	5‐of‐5 in silico tools (FATHMM, LRT, mutation assessor, mutation taster, and SIFT) predict a damaging effect
PP4	Supporting	Biochemically confirmed hyperinsulinism (critical sample) with markedly elevated glucose requirement (GIR up to 17 mg/kg/min IV plus 9 mg/kg/min enteral); diffuse pancreatic uptake on ^18^F‐DOPA PET‐CT, consistent with the diffuse disease pattern expected for a homozygous *ABCC8* variant; early‐onset presentation (2 h of life) typical of KATP‐channel‐related congenital hyperinsulinism

*Note:* The identified variant has been reported in dbSNP database with an Identification Number rs367850779.

### 2.5. Outcome

At 15 months of age, the patient remains under regular follow‐up with no new episodes of hypoglycemia. The diazoxide dose has been naturally weaned to 5 mg/kg/day as the infant gained weight. The current clinical plan is to continue home glucose monitoring and slowly taper the diazoxide with the intention of eventually discontinuing the medication. He is growing normally, hitting all his milestones, and keeping healthy blood sugar levels. Neurodevelopment assessments were conducted routinely at each follow‐up visit. These assessments confirmed that the infant met all motor, cognitive, communication, and social milestones appropriately for his age. A comprehensive timeline of clinical diagnosis and management is detailed in (Table 4).

**Table 4:** Clinical timeline of diagnosis and management of the patient.

Day/Time	Event	Management
Birth	Large for gestational age infant (5.26 kg) born at 34 + 5 weeks.	Monitored via standard protocol (at 1 h of life, then prefeed every 2 h).
Hour 1–2	Stable GRBS	Hour 1: GRBS 46 mg/dL. Feeds started. Hour 2: GRBS 50 mg/dL after feeding.
Hour 4	Hypoglycemia onset. Infant was lethargic and jittery.	GRBS dropped to 16 mg/dL. Action: Immediate IV dextrose bolus given; monitoring increased to hourly checks.
Post‐hour 4	GIR escalation due to persistent hypoglycemia.	IV GIR increased beyond 12 mg/kg/min, triggering a critical sample draw at 52 HOL.
Post‐hour 52	Hyperinsulinemia detected	Lab results: Insulin and C‐peptide (Table 2) Octreotide initiated: Started at 5 mcg/kg/day SC (due to diazoxide unavailability).
NICU stay (titration)	Medical stabilization and medication escalation.	Octreotide escalation: Increased up to 25 mcg/kg/day SC in divided doses. Diazoxide initiated: Started at 5 mg/kg/day PO (compounded into syrup from capsules). Diazoxide escalation: Gradually increased to 15 mg/kg/day PO. Adjunct drug: Hydrochlorothiazide added at 5 mg/kg/day to prevent fluid overload from diazoxide.
NICU stay (feeds)	Oral GIR introduction and weaning.	Due to poor IV access, an oral GIR up to 9 mg/kg/min was introduced via a syringe pump. Once stabilized on diazoxide, oral GIR, and octreotide were both tapered and stopped.
Month 2	Seizure episode and discharge.	Seizures occurred despite normal glucose levels. Action: Started on levetiracetam. Discharged on diazoxide, hydrochlorothiazide, and levetiracetam.
Month 5	Diazoxide unavailability causes relapse.	Missed doses led to a hypoglycemic seizure. Action: Readmitted for IV GIR and then restabilized on diazoxide at 15 mg/kg/day.
Month 8	Genetic and imaging follow‐up in India.	Confirmed homozygous pathogenic mutation in the *ABCC8* gene. PET scan showed diffuse uptake in bulky pancreas.
Month 15	—	Neurodevelopment is completely normal. Current dose: Diazoxide self‐weaned with weight gain down to 5 mg/kg/day. Plan to slowly stop it soon.

## 3. Discussion

CHI is an autosomal disorder characterized by refractory hypoglycemia in infants due to excessive insulin secretion by pancreatic beta cells [18]. It is commonly associated with defects in the beta‐cell KATP‐channel genes, *KCNJ11* and *ABCC8*, which encode the channel subunits Kir6.2 and sulfonylurea receptor (SUR1) [8]. A homozygous *ABCC8* mutation was identified in our patient.

Clinical manifestations include floppiness, jitteriness, poor feeding, lethargy, and irritability, which can progress to seizures, coma, irreversible brain injury, and neonatal death if untreated [19]. Hypertrophic cardiomyopathy and hepatomegaly from glycogen accumulation, possibly related to fetal hyperinsulinemia (HI), may also occur [20]. Mild hepatomegaly was noted in our patient.

CHI has two histological subtypes: diffuse, affecting the entire pancreas (inherited autosomal recessively or dominantly), and focal, involving only a portion and usually sporadic [2]. The ^18^F‐DOPA‐PET scan has up to 96% accuracy in distinguishing the two [4, 21]. Diffuse HI arises from biallelic recessive mutations, whereas focal HI results from paternal transmission of a monoallelic mutation followed by somatic loss of maternal 11p15.1 [13]. In our case, the PET scan revealed a uniformly enlarged pancreas, consistent with diffuse involvement.

The primary goal of treatment is achieving normoglycemia and restoring ketone body production to provide alternative brain energy [21]. Diazoxide is the first‐line therapy (5–15 mg/kg/day orally), acting as a KATP‐channel agonist to inhibit insulin release. It is initiated once HI is confirmed and GIR requirements continue to rise even after a week. Diazoxide is metabolized in the liver and excreted in the kidneys, and protein‐bound; the dose is started at 3 mg/kg/day in infants with hepatic dysfunction and hypoalbuminemia with close monitoring. Due to fluid retention leading to serious complications like pericardial effusion, it is administered alongside thiazide diuretics [22]. Other common side effects of diazoxide include hypertrichosis, fluid overload or edema, electrolyte imbalances, necrotizing enterocolitis, and pulmonary hypertension, which typically resolve after the drug is discontinued [23, 24]. Consensus guidelines recommend an echocardiogram about a week after starting diazoxide [24]. In this case, hydrochlorothiazide was added to avoid fluid retention, electrolytes were monitored, and echocardiography was done prior to starting diazoxide and on follow‐up. No known side effects were seen in the patient.

In diazoxide‐unresponsive cases or when the drug is unavailable, glucagon and/or octreotide alongside high‐concentration glucose infusions can be used [4]. Octreotide (5–35 mcg/kg/day) serves as the second‐line medical therapy [22]. Chandran et al. [25] has shown improvement of an infant with octreotide when GIR continued to rise on diazoxide [25]. ^18^F‐DOPA‐PET guides surgical planning. Focal lesions can be cured by partial pancreatectomy or enucleation, while diffuse lesions may require subtotal (≈85%) or near‐total (≈95%–97%) resection, carrying risks of relapse, exocrine pancreatic insufficiency, and later diabetes mellitus [3, 26, 27]. In our patient, the diffuse lesion responded to diazoxide, avoiding surgical intervention entirely.

Homozygous *ABCC8* mutations typically cause diazoxide nonresponsiveness, because they disrupt the structural integrity or trafficking of SUR1, causing the target site of diazoxide to become nonfunctional. Most reported cases, including De Franco et al. [28] require pancreatic surgery. However, our patient is a rare exception, with a positive diazoxide response, which suggests he might still have some working KATP channels similar to partial responders in the homozygous *ABCC8* mutation mentioned by Kiff et al. [1, 29]. Arya et al. [30] have also reported a case where the child was responsive to diazoxide; however, the child had a compound heterozygous mutation. Takasawa et al. [31] similarly documented a 13‐month old with a biallelic *ABCC8* mutation presenting with hypoglycemic seizure who responded to diazoxide.

Genotype–phenotype correlation is significant, as accurate and timely prediction of phenotype based on genotype assists with planning treatment strategy; however, in *ABCC8*‐related CHI, it is further complicated by variable penetrance and expressivity, even among carriers of the identical variant. Dominantly acting *ABCC8* mutations have been shown to produce phenotypes ranging from asymptomatic hypoglycemia to severe, diazoxide‐unresponsive disease within the same family, and marked clinical heterogeneity, including differing diazoxide responsiveness, has similarly been documented among relatives homozygous for the same variant [31, 32]. Modifier genes, mosaicism, or tissue‐specific differences in channel expression likely contribute to the variability observed even in identical genotypes. The largest available genotype–phenotype series found that recessive *ABCC8*/*KCNJ11* mutations were strongly, but not universally, predictive of diazoxide unresponsiveness, accentuating that genotype alone cannot reliably forecast clinical course in every case, as illustrated by our patient [28].

Recessive KATP‐channel activity in biallelic *ABCC8* mutations can be explained by the distinction between trafficking and gating defects. Trafficking mutations impair transport of the SUR1‐Kir6.2 complex to the plasma membrane and generally abolish diazoxide response, whereas gating mutations allow the channel to reach the cell surface but impair its opening in response to MgADP and diazoxide. However, certain missense variants of this latter type can leave a small residual population of surface channels capable of at least partial pharmacological activation, providing the substrate for diazoxide responsiveness as seen in this patient [33]. Variability in diazoxide response may also be related to the proximity of the mutation site to the ATP‐binding sites. Chang et al. [34] reported eight cases of *ABCC8* mutation, out of which three were diazoxide responsive; all responsive cases had a mutation in the *NBD2* domain of the *ABCC8* gene, though clinical manifestations and prognosis weren’t identical even among those sharing the same mutation.

In diazoxide‐responsive cases, the GIR is gradually weaned until full oral feeds are established [22]. A 6‐h age‐appropriate safety fast study confirms the infant’s ability to maintain normoglycemia during an inadvertent fast at home, after which home glucose monitoring is continued. Diazoxide is allowed to self‐wean as the weight increases with stable plasma glucose levels [22]. Diazoxide is metabolized by CYP1A2, and its activity is negligible at birth. After ~8 months of age, the CYP1A2 activity matures and approaches adult level, and renal function is well‐established, lowering the systemic exposure at a fixed weight‐based dose over time. It also allows gradual reduction of weight‐based dosing [35]. In this case, the parents were taught to measure capillary blood glucose via a glucometer. Plasma glucose and electrolytes were measured at follow‐up visits. Diazoxide was allowed to self‐wean with the weight gain of the patient. As highlighted in a previous report by Virú‐Loza et al. [36], our experience further supports the importance of ensuring diazoxide availability and its inclusion in the national essential medicines list to timely manage CHI.

During management of this patient, an important clinical challenge was the utilization of an enteral GIR via an OG tube due to inaccessible vascular access. Theoretically, administration of enteral nutrition or carbohydrates causes secretion of incretins—glucose‐dependent insulinotropic polypeptide (GIP) and glucagon‐like peptide‐1 (GLP‐1)—which stimulate insulin secretion by activation of adenylate cyclase and generation of cAMP. Exendin‐(9‐39) is a specific GLP‐1 receptor antagonist, which decreases cAMP levels and inhibits insulin secretion [37]. In CHI, where there is excessive insulin secretion, the incretin effect can aggravate HI. However, a study was done at the Children’s Hospital of Philadelphia (CHOP) by Vajravelu et al. [6] has explained that continuous intragastric dextrose has been shown to be safe and well‐tolerated in CHI, with GIR ≤10 mg/kg/min to avoid high osmotic load and intolerance. Our patient received an enteral GIR of up to 9 mg/kg/min via an OG tube. The enteral dextrose administration was done with continuous GRBS monitoring for possible hypoglycemia, as the incretin pathways may amplify insulin secretion.

This case demonstrates successful management of CHI in a preterm neonate with a homozygous *ABCC8* mutation, highlighting a positive response to diazoxide despite expectations of nonresponsiveness. The use of oral glucose infusion to stabilize blood glucose illustrates an effective, innovative approach in a challenging clinical scenario. Genotype alone should not determine therapeutic decisions, and clinical response to diazoxide remains an essential component of individualized management.

### 3.1. Limitation

1. Medication supply‐chain instability: The lack of domestic availability of diazoxide, the primary first‐line therapy for CHI, resulted in initial management delays. Furthermore, the subsequent shortage of the drug led to a severe hypoglycemic seizure at 5 months, highlighting the life‐threatening risks associated with the inconsistent supply of specialized medications for rare metabolic disorders.
2. Although MRI brain is the gold standard for identifying hypoglycemia‐induced parieto‐occipital injury, parents declined MRI brain, genetic testing, and DOPA scan due to financial constraints.
3. Incomplete genotypic mapping: While the proband’s homozygous mutation was confirmed, paternal and maternal genetic testing could not be performed due to financial limitations. This prevented a formal segregation analysis to confirm the inheritance pattern (e.g., autosomal recessive vs. uniparental disomy), which is essential for providing the family with precise recurrence‐risk counseling.

### 3.2. Key Takeaway for Clinicians

This case highlights that a trial of diazoxide should be considered even in patients with homozygous *ABCC8* mutations, as variable responsiveness may occur. In resource‐limited settings, pragmatic strategies such as enteral glucose infusion and stepwise medical management can help achieve glycemic stability when advanced therapies are not readily available.

## 4. Conclusion

Early recognition and prompt management of CHI are critical to prevent neurological injury. This case demonstrates rare diazoxide responsiveness despite a homozygous *ABCC8* mutation. This case suggests that a carefully monitored therapeutic trial of diazoxide may still be reasonable in selected patients with homozygous *ABCC8* mutations before concluding complete medical unresponsiveness, particularly in resource‐limited settings.

**Nomenclature**
CHI: Congenital hyperinsulinism
HI: Hyperinsulinemia
LGA: Large for gestational age
NICU: Neonatal intensive care unit
GIR: Glucose infusion rate
NPO: Nil per oral
PET: Positron emission tomography
^18^FDG DOPA: 2‐Deoxy‐2‐[fluorine‐18]fluoro‐D‐glucose
KATP: ATP‐sensitive potassium
GRBS: Glucose random blood sugar
IV: Intravenous
OG: Orogastric
USG: Ultrasonography
MDCT: Multidetector computed tomography
PDA: Patent ductus arteriosus.

## Author Contributions

**Grisha Gurung**: writing – original draft. **Prerana Kansakar**: supervision, conceptualization, editing. **Grisha Gurung, Sadikshya Bhandari, Kriti Shrestha, Bhumika G. C., Shreya Thapa, and Rasik Acharya**: editing.

## Funding

This article has not received any funding or grant.

## Disclosure

All authors have read and approved the final version of the manuscript. Grisha Gurung and all the authors had full access to all of the data in this study and take complete responsibility for the integrity of the data and the accuracy of the data analysis.

## Consent

The consent was taken from the baby’s parents for the publication of this case report and the publication of the genetic/imaging data.

## Conflicts of Interest

The authors declare no conflicts of interest.

## Patient Perspective

Despite diagnostic challenges that required travel to India, successful treatment and normal development brought the family relief.

## Data Availability

The data that support the findings of this study are available from the corresponding author upon reasonable request.

## References

[1] Kiff S. , Babb C. , and Guemes M. , et al.Partial Diazoxide Responsiveness in a Neonate With Hyperinsulinism due to Homozygous ABCC8 Mutation, Endocrinology, Diabetes & Metabolism Case Reports. (2019) 2019, 10.1530/EDM-18-0120. PMC6373619 30753133

[2] Hewat T. I. , Johnson M. B. , and Flanagan S. E. , Congenital Hyperinsulinism: Current Laboratory-Based Approaches to the Genetic Diagnosis of a Heterogeneous Disease, Frontiers in Endocrinology. (2022) 13, 10.3389/fendo.2022.873254, 889678. PMC9302115 35872984

[3] Josefina S. , Pattillo J. C. , Orellana P. , and Godoy C. , Hiperinsulinismo Congénito Del Recién Nacido: A Propósito De Un Caso Clínico, Revista chilena de pediatría. (2017) 88, no. 3, 377–382, 10.4067/S0370-41062017000300010. 28737197

[4] Hasbaoui B. E. , Elyajouri A. , Abilkassem R. , and Agadr A. , Congenital Hyperinsulinsim: Case Report and Review of Literature, Pan African Medical Journal. (2020) 35, 10.11604/pamj.2020.35.53.16604. PMC7250230 32537058

[5] Rozance P. J. , Connor R. F. , Management and Outcome of Neonatal Hypoglycemia, UpToDate, 2025, Wolters Kluwer.

[6] Vajravelu M. , Congdon M. , and Mitteer L. , et al.Continuous Intragastric Dextrose: A Therapeutic Option for Refractory Hypoglycemia in Congenital Hyperinsulinism, Hormone Research in Paediatrics. (2018) 91, no. 1, 62–68, 10.1159/000491105. PMC6661174 30086540

[7] NCBI , ClinVar, Nih.gov.2025, 2026, NM_000352.6(ABCC8): C.3641G>a (p.Arg1214Gln) AND Not Provided - https://www.ncbi.nlm.nih.gov/clinvar/RCV001388601.11/.

[8] Bellanné-Chantelot C. , Saint-Martin C. , and Ribeiro M. J. , et al.ABCC8 and KCNJ11 Molecular Spectrum of 109 Patients With Diazoxide-Unresponsive Congenital Hyperinsulinism, Journal of Medical Genetics. (2010) 47, no. 11, 752–759, 10.1136/jmg.2009.075416. 20685672

[9] Nestorowicz A. , Glaser B. , and Wilson B. A. , et al.Genetic Heterogeneity in Familial Hyperinsulinism, Human Molecular Genetics. (1998) 7, no. 7, 1119–1128, 10.1093/hmg/7.7.1119. 9618169

[10] Shyng S. L. , Ferrigni T. , and Shepard J. B. , et al.Functional Analyses of Novel Mutations in the Sulfonylurea receptor 1 Associated With Persistent Hyperinsulinemic Hypoglycemia of Infancy, Diabetes. (1998) 47, no. 7, 1145–1151, 10.2337/diabetes.47.7.1145. 9648840

[11] Henwood M. J. , Kelly A. , and Macmullen C. , et al.Genotype-Phenotype Correlations in Children With Congenital Hyperinsulinism due to Recessive Mutations of the Adenosine Triphosphate-Sensitive Potassium Channel Genes, The Journal of Clinical Endocrinology & Metabolism. (2005) 90, no. 2, 789–794, 10.1210/jc.2004-1604. 15562009

[12] Suchi M. , MacMullen C. , Thornton P. S. , Ganguly A. , Stanley C. A. , and Ruchelli E. D. , Histopathology of Congenital Hyperinsulinism: Retrospective Study With Genotype Correlations, Pediatric and Developmental Pathology. (2003) 6, no. 4, 322–333, 10.1007/s10024-002-0026-9. 14692646

[13] Snider K. E. , Becker S. , and Boyajian L. , et al.Genotype and Phenotype Correlations in 417 Children With Congenital Hyperinsulinism, The Journal of Clinical Endocrinology & Metabolism. (2013) 98, no. 2, E355–E363, 10.1210/jc.2012-2169. PMC3565119 23275527

[14] Yan F. F. , Lin Y. W. , MacMullen C. , Ganguly A. , Stanley C. A. , and Shyng S. L. , Congenital Hyperinsulinism Associated ABCC8 Mutations That Cause Defective Trafficking of ATP-Sensitive K+ Channels: Identification and Rescue, Diabetes. (2007) 56, no. 9, 2339–2348, 10.2337/db07-0150. PMC2225993 17575084

[15] Mohnike K. , Wieland I. , and Barthlen W. , et al.Clinical and Genetic Evaluation of Patients With KATP Channel Mutations From the German Registry for Congenital Hyperinsulinism, Hormone Research in Paediatrics. (2014) 81, no. 3, 156–168, 10.1159/000356905. 24401662

[16] Demirbilek H. , Shah P. , and Arya V. B. , et al.Long-Term Follow-Up of Children With Congenital Hyperinsulinism on Octreotide Therapy, The Journal of Clinical Endocrinology & Metabolism. (2014) 99, no. 10, 3660–3667, 10.1210/jc.2014-1866. 24937539

[17] Brady C. , Palladino A. A. , and Gutmark-Little I. , A Novel Case of Compound Heterozygous Congenital Hyperinsulinism Without High Insulin Levels, International Journal of Pediatric Endocrinology. (2015) 2015, no. 1, 10.1186/s13633-015-0012-4, 16. PMC4502541 26180531

[18] Hussain K. , Blankenstein O. , De Lonlay P. , and Christesen H. T. , Hyperinsulinaemic Hypoglycaemia: Biochemical Basis and the Importance of Maintaining Normoglycaemia During Management, Archives of Disease in Childhood. (2007) 92, no. 7, 568–570, 10.1136/adc.2006.115543. PMC2083756 17588969

[19] Otonkoski T. , Näntö-Salonen K. , and Seppänen M. , et al.Noninvasive Diagnosis of Focal Hyperinsulinism of Infancy With [18F]-DOPA Positron Emission Tomography, Diabetes. (2006) 55, no. 1, 13–18, 10.2337/diabetes.55.01.06.db05-1128. 16380471

[20] Aynsley-Green A. , Hussain K. , and Hall J. , et al.Practical Management of Hyperinsulinism in Infancy, Archives of Disease in Childhood - Fetal and Neonatal Edition. (2000) 82, no. 2, F98–F107, 10.1136/fn.82.2.F98. PMC1721064 10685981

[21] Sweet C. B. , Grayson S. , and Polak M. , Management Strategies for Neonatal Hypoglycemia, The Journal of Pediatric Pharmacology and Therapeutics. (2013) 18, no. 3, 199–208, 10.5863/1551-6776-18.3.199. PMC3775554 24052783

[22] Chandran S. , Rajadurai V. S. , Hussain K. , and Yap F. , Physiological and Phased Approach to Newborns at-Risk of Hyperinsulinemic Hypoglycemia: A Neonatal Perspective, Journal of Clinical Neonatology. (2019) 8, no. 4, 193–202, 10.4103/jcn.JCN_37_19.

[23] Desai J. , Key L. , Swindall A. , Gaston K. , and Talati A. J. , The Danger of Diazoxide in the Neonatal Intensive Care Unit, Therapeutic Advances in Drug Safety. (2021) 12, 10.1177/20420986211011338, 20420986211011338. PMC8135194 34046157

[24] Newman-Lindsay S. , Lakshminrusimha S. , and Sankaran D. , Diazoxide for Neonatal Hyperinsulinemic Hypoglycemia and Pulmonary Hypertension, Children. (2023) 10, no. 1, 10.3390/children10010005, 5. PMC9856357 36670556

[25] Chandran S. , Tun W. L. , Htay P. T. , and Hussain K. , Spontaneous Resolution of Congenital Hyperinsulinism With Octreotide Therapy, Indian Pediatrics. (2020) 57, no. 5, 474–475, 10.1007/s13312-020-1829-3. 32444525

[26] Shah P. , Demirbilek H. , and Hussain K. , Persistent Hyperinsulinaemic Hypoglycaemia in Infancy, Seminars in Pediatric Surgery. (2014) 23, no. 2, 76–82, 10.1053/j.sempedsurg.2014.03.005. 24931352

[27] Petraitienė I. , Barauskas G. , and Gulbinas A. , et al.Congenital Hyperinsulinism, Medicina. (2014) 50, no. 3, 190–195, 10.1016/j.medici.2014.08.006. 25323548

[28] De Franco E. , Saint-Martin C. , and Brusgaard K. , et al.Update of Variants Identified in the Pancreatic β-Cell KATP Channel Genes KCNJ11 and ABCC8 in Individuals With Congenital Hyperinsulinism and Diabetes, Human Mutation. (2020) 41, no. 5, 884–905, 10.1002/humu.23995. PMC7187370 32027066

[29] Rozenkova K. , Guemes M. , Shah P. , and Hussain K. , The Diagnosis and Management of Hyperinsulinaemic Hypoglycaemia, Journal of Clinical Research in Pediatric Endocrinology. (2015) 7, no. 2, 86–97, 10.4274/jcrpe.1891. PMC4563192 26316429

[30] Arya V. B. , Aziz Q. , Nessa A. , Tinker A. , and Hussain K. , Congenital Hyperinsulinism: Clinical and Molecular Characterisation of Compound Heterozygous ABCC8 Mutation Responsive to Diazoxide Therapy, International Journal of Pediatric Endocrinology. (2014) 2014, no. 1, 10.1186/1687-9856-2014-24, 2. PMC4290134 25584046

[31] Takasawa K. , Miyakawa Y. , and Saito Y. , et al.Marked Clinical Heterogeneity in Congenital Hyperinsulinism due to a Novel Homozygous ABCC8 Mutation, Clinical Endocrinology. (2021) 94, no. 6, 940–948, 10.1111/cen.14443. 33595839

[32] Flanagan S. E. , Kapoor R. R. , and Banerjee I. , et al.Dominantly Acting ABCC8 Mutations in Patients With Medically Unresponsive Hyperinsulinaemic Hypoglycaemia, Clinical Genetics. (2011) 79, no. 6, 245–252, 10.1111/j.1399-0004.2010.01476.x. PMC3375476 20573158

[33] ElSheikh A. and Shyng S.-L. , KATP Channel Mutations in Congenital Hyperinsulinism: Progress and Challenges Towards Mechanism-Based Therapies, Frontiers in Endocrinology. (2023) 14, 10.3389/fendo.2023.1161117, 1161117. PMC10086357 37056678

[34] Chang G. , Ying L. , and Zhang Q. , et al.Genetic Variants of ABCC8 and Clinical Manifestations in Eight Chinese Children With Hyperinsulinemic Hypoglycemia, BMC Endocrine Disorders. (2024) 24, no. 1, 10.1186/s12902-023-01527-8, 8. PMC10785495 38212772

[35] Wong T. , Chan D. , and Chua C. , et al.From Standard to Individualized Diazoxide Therapy in Congenital Hyperinsulinism: A Narrative Review, Frontiers in Pharmacology. (2026) 17, 10.3389/fphar.2026.1781424, 1781424. PMC13071010 41982675

[36] Virú-Loza M. A. , Bermudez-Paredes F. , and Arauco-Carhuas I. M. , Severe Transient Neonatal Hyperinsulinism: First Peruvian Case Series, Sage Open Medical Case Reports. (2025) 13, 10.1177/2050313X251347787. PMC12166237 40520083

[37] Demirbilek H. and Hussain K. , Congenital Hyperinsulinism: Diagnosis and Treatment Update, Journal of Clinical Research in Pediatric Endocrinology. (2018) 9, no. 2, 69–87, 10.4274/jcrpe.2017.S007. PMC5790328 29280746

